# Phenotype–genotype correlation in children with familial Mediterranean fever in Morocco

**DOI:** 10.5339/qmj.2024.41

**Published:** 2024-11-25

**Authors:** Manal Souali, Asmaa Sakhi, Ahmed Aziz Bousfiha, Kenza Bouayed

**Affiliations:** 1 Laboratory of Clinical Immunology, Inflammation and Allergy (LICIA), Hassan II University - Faculty of Medicine and Pharmacy of Casablanca, Morocco *Email: soualimanal@gmail.com; 2 Pediatric Rheumatology and Internal Medicine Unit, A. Harouchi Mother and Child Hospital, Ibn Rochd University Hospital Center, Casablanca, Morocco; 3 Clinical Immunology and Infectious diseases Unit, A. Harouchi Mother and Child Hospital, Ibn Rochd University Hospital Center, Casablanca, Morocco

**Keywords:** Autoinflammatory diseases, children, familial Mediterranean fever, genetics

## Abstract

**Background:**

Familial Mediterranean fever (FMF) is an autosomal recessive disease caused by mutations in the MEFV gene and is characterized by recurrent febrile episodes of abdominal pain, chest pain, and joint involvement. We aim to study the clinical and genetic features of FMF in Moroccan children and to establish a phenotype–genotype correlation in this group of patients.

**Methods:**

A total of 35 patients were included in this study. Genetic analysis of exon 10 of the *MEFV* gene was performed in 33 patients. To establish a phenotype–genotype correlation, we statistically compared clinical features between patients with and without the *M694V* mutation.

**Results:**

Abdominal pain was observed in 82.9% of our patients, followed by fever (74.3%), arthralgia (85.7%), arthritis (42.8%), chest pain (34.3%), and IgA vasculitis (20%). Genetic analysis showed a predominance of the *M694V* mutation (62.5%), followed by *A744S* (11.4%) and K695R (5.7%). The presence of the *M694V* genotype was found to be significantly associated with a high frequency of arthralgia and arthritis. A significant association was found with an earlier age of onset in the absence of the *M694V* mutation.

**Conclusion:**

Joint involvement is more common in the *M694V* genotype, and the genetic profile shows different results compared to neighboring countries.

## 1. Introduction

Familial Mediterranean fever (FMF, OMIM #249100) is the most common monogenic autoinflammatory disease in the world. It is characterized by recurrent self-limited febrile episodes lasting about 1–3 days.^1^ It is a pediatric disease as the first manifestations appear at approximately 4 years and in 80% of cases before the age of 20 years. Patients are usually symptom-free between attacks; however, subacute or chronic symptoms may occur.^2^ FMF has been historically classified into two phenotypes: Phenotype I, with frequent typical symptoms and clinical signs of FMF, and less common Phenotype II, with amyloidosis as the only clinical manifestation in an asymptomatic individual. A third phenotype has been suggested following the identification of the *MEFV* gene, with the presence of biallelic pathogenic variants with no clinical signs nor increased inflammatory markers.^3^ Systematic reviews have defined a wide range of symptoms associated with FMF; the well-known classical manifestations are fever, serositis with abdominal and chest pain, arthritis, and erysipelas-like erythema.^4^

FMF affects over 120,000 people. It occurs mainly in ethnic groups surrounding the Mediterranean basin, most often in Jews, Turks, Armenians, and Arabs, and can affect 1/500 people. Turkey is considered the country with the highest number of FMF patients, followed by Armenia and Israel. FMF is also present in North Africa, Greece, Crete, France, Germany, and the United States.^3,5^ In Arab and Maghreb countries, the exact prevalence of FMF is still underestimated due to a deep lack of data. To date, 1 in 2600 Arab children is affected by FMF, with a genetic frequency of 1 in 50.^6^

FMF is caused by “gain of function” mutations in the *MEFV* gene, which is located on the short arm of chromosome 16p13.3, composed of 10 exons and coding for a 781-amino acid protein called “pyrin or marenostrin,” an important regulator of the innate immune response.^7^ Classical FMF is an autosomal recessive disease caused by homozygous or compound heterozygous mutations in the *MEFV* gene. However, an autosomal dominant inherited form has also been described.^8^ To date, a total of 391 different sequence variants of the *MEFV* gene have been recorded in the “Infevers” database according to the International Society of Systemic Auto-Inflammatory Diseases “ISSAID",^9^ with the mutation *M694V* being the most encountered and pathogenic.^2^ In North Africa, the disease carriage rate has been estimated at 1%.^10^ However, data on the molecular spectrum of FMF in the Moroccan population are still lacking. The extreme variability in the clinical presentation and severity of FMF has prompted researchers to analyze genotype–phenotype correlations. We aim to assess the clinical features of FMF in Moroccan children and to establish a phenotype–genotype correlation in this group of patients.

## 2. Patients and Methods

### 2.1. Patients

Patients of this study were referred from the Pediatric Rheumatology unit of University Mother-Child Hospital, University Hospital Center Ibn Rochd of Casablanca, and private practices from local pediatric rheumatology experts. From 2008 to 2022, a total of 35 children clinically suspected of having FMF were referred. The diagnosis was based on the Pediatric Rheumatology INternational Trials Organization “PRINTO"/Eurofever 2019 criteria.^11^ Data collected from the medical records included personal information, clinical signs, and results of biological and genetic tests and therapeutic management. The study was approved by the Ibn Rochd University Hospital Ethics Committee and written informed consent was signed by the legally authorized representatives of the patients for the genetic analysis prior to their inclusion in the study for publication.

### 2.2. PCR amplification and sequencing

Genetic analysis was performed only in 33 of 35 patients of our series due to loss of follow-up in two patients. Genomic deoxyribonucleic acid (DNA) was isolated from peripheral blood leucocytes according to standard procedures.^12^ For each patient, genetic testing consisted of sequencing exon 10 of the *MEFV* gene, followed by exon 2 subsequently in case of no mutations detected in the first one. Genetic analysis of exon 3 in one patient was performed outside our institution. Exons were amplified by polymerase chain reaction (PCR) and screened for mutations through direct sequencing, using two pairs of corresponding primers detailed below.

Exon 10: F: 5′-GAGGTGGAGGTTGGAGACAA-3′

     R: 5′-TGACCACCCACTGGACAGAT-3′

Exon 2: F: 5′-GCCTGAAGACTCCAGACCACCCCG-3′

    R: 5′-AGGCCCTCCGAGGCCTTCTCTCTG-3′

PCR conditions were as follows: initial denaturation at 95°C for 5 min, 35 cycles at 94°C for 30 s and 60°/59°C (exons 10 and 2, respectively) for 45 s and 72°C for 1 min, and a final extension at 72°C for 5 min. Purified PCR products were sequenced along both strands using the Sanger targeted DNA sequencing method on ABI 3500, Thermo Fisher, with an ABI Big Dye terminator v1.1 kit (Applied Biosystems, Foster City, CA) according to the manufacturer’s instructions. The *MEFV* mutation analysis was performed for the common mutations including *M680I*, *M680L*, *I692del*, *M694V*, *M694I*, *L695A*, *V726A*, *K695R*, and *A744S* in exon 10, and *R202Q*, *R761H*, *G196W*, and *E148Q* in exon 2. The exon 2 analysis could not be performed in all patients negative for exon 10 as it was available in our hospital for a specific study and only three patients could benefit of it.

### 2.3. Statistical analysis

Values were presented as means with minimum–maximum ranges. Statistical analysis was performed using the Statistical Package for the Social Sciences (SPSS) version 26.0. For our small sample size of patients, we used the Fisher test for differences between groups. A *p* value <0.05 was accepted as statistically significant.

### 2.4. Ethics approval and consent for participation and publication

Written informed consent for participation and publication was obtained from the legally authorized representatives of the patients and held in their hospital records. For studies involving human participants, all procedures performed were approved by the institutional ethics committee of the University Hospital Center Ibn Rochd under the reference 2021/DOEHRSI/31 and file N° 03/21, and conformed with its ethical standards as well as with the 1964 Helsinki Declaration and its later amendments or comparable ethical standards. The consent for publication was obtained from the children’s parents and legally authorized representative.

## 3. Results

### 3.1. General characteristics of our patients

All patients were of Moroccan origin, except two patients; a Syrian girl and a French-Vietnamese boy. Among the 35 children, 51.4% were females and 48.6% were males with a sex ratio of 1.06. The mean age of symptoms onset was 6 years (10 months–16 years), and the mean age at first consultation was 7 years and 4 months (18 months–16 years and 2 months). Consanguinity was reported in 54.3% of cases and unknown in one case of an adopted child. Seven of our patients had less than 1 attack per month, 15 had a frequency of 1 attack to 2 per month, and 8 had more than 2 attacks per month. Moreover, one patient had a prolonged fever of 2 months, while the frequency remained unknown in four patients because of difficult medical history.

### 3.2. Clinical features of the patients

**Figure 1.**
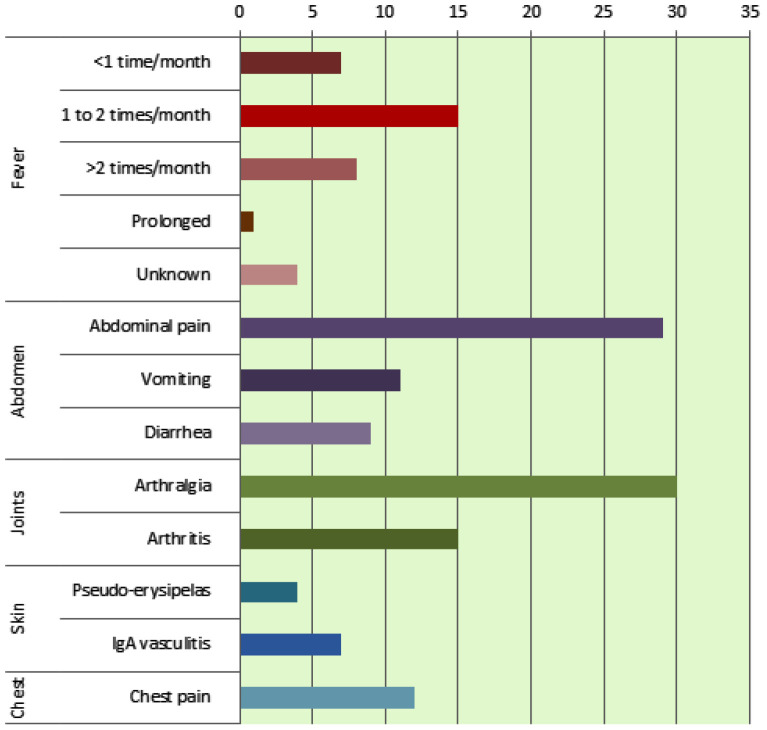
The main clinical symptoms of patients in our study.

The main clinical characteristics of our patients are shown in Figure 1. The presence of high-grade fevers (39–40°C) was found in 74.3% of cases, abdominal pain in 82.9% associated with diarrhea and vomiting (25.7% and 31.4%, respectively), arthralgia in 85.7%, arthritis in 42.8%, and chest pain in 34.3%. Oral aphthous was found in 14.3% of patients, erysipelas-like erythema in 11.4%, myalgia in 11.4%, headache in 14.3%, and lymphadenopathy in 20% each. Henoch–Schönlein purpura (Immunoglobulin A “IgA” vasculitis) was found in 20% each and was diagnosed on the basis of a clinical triad of vascular purpura, joint involvement, and abdominal pain. No amyloidosis was reported in our series. All patients were treated with 1 mg/day colchicine as an initial dose after being included in the study. The response to the treatment was assessed from the first weeks of administration: complete remission was noted in 71.4% of cases, and partial response in 17.1% with persistent abdominal and joint pain and/or high inflammatory syndrome. Three patients were lost to follow-up and one case of unexplained sudden death occurred. Complete remission was defined as the absence of clinical signs and normalization of inflammatory parameters. Partial remission was defined as decreasing but persistent mild symptoms, spacing of episodes, or decreasing but persistent mild inflammatory syndrome.

### 3.3. Mutations detected in the MEFV gene

The analysis of the *MEFV* gene exons revealed the presence of mutations in 32 of 33 patients and their absence in one patient, in whom the diagnosis of FMF was however based on the presence of increased inflammatory markers and a typical FMF clinical picture according to PRINTO/Eurofever defined criteria. The patient considered to have FMF without genetic confirmation had the typical symptoms of the disease and was tested only for exon 10 mutations. Among those 32 patients, 46.8% were homozygous, 50% were heterozygous, and the last one was compound heterozygous (3%). The genetic analysis allowed the identification of eight different *MEFV* mutations (Table 1) and showed a predominance of the *M694V* mutation, at 40% in the homozygous state and 17.1% in the heterozygous state, followed by *A744S* (11.4%) and *K695R* (5.7%). The remaining mutations *M694I*, *E148Q*, *R202Q*, and *P369S*, and the variants *R408Q* and *G196W* were much less represented.

### 3.4. Genotype–phenotype correlation

In order to evaluate the genotype–phenotype correlation, 32 patients with genetically confirmed mutations in *MEFV* were divided into three groups according to the presence of the *M694V* mutation, reported to be the most pathogenic and frequent worldwide, and most frequent in our study: group I with *M694V* on both alleles (*M694V*/*M694V*), group II with *M694V* on a single allele (*M694V*/other), and group III with *M694V* on none of the alleles (*other/other*). This latter includes different mutations identified in our study other than *M694*, which nevertheless present varying degrees of pathogenicity in FMF. Fourteen clinical features of FMF were compared in these patients, as represented in Table 2. However, no statistically significant differences were observed between these groups regarding the male/female ratio, fever, abdominal and chest pain, headache, myalgia, erysipelas-like erythema, IgA vasculitis, and response to treatment. Nevertheless, statistically significant differences were noted between groups I and III regarding joint involvement (arthralgia and arthritis) and frequency rate, and groups II and III regarding the age of symptoms onset.

## 4. Discussion

Genetic research on FMF has revealed great diversity in the distribution of *MEFV* gene mutations in different populations, and even in the same population of different regions of the country. To date, data from the literature on genotype–phenotype correlations in FMF remain inconclusive.^13^ The frequency of FMF was similar in both genders with a sex ratio of 1.06 in our patients. This result is similar to previous studies, reporting an approximately equal prevalence in both sexes among Arabs,^14,15^ whereas in other ethnic groups, the prevalence could differ.^2^ Family history of clinical FMF was reported in 34.3%, and consanguinity was detected in 54.3% of our patients. Numerous studies have reported family history and parental consanguinity at different rates worldwide, specifically in Arab countries.^8,15,16^

The main clinical symptoms of FMF have been reported previously; abdominal pain is the most common feature in approximately 82–96% of FMF patients.^17^ In our patients, abdominal pain was observed in 82.9%, followed by fever (74.3%). Joint involvement is the second most common hallmark of FMF associated with fever and was detected in all of our patients. The frequency of joint involvement varies among different ethnic groups; arthritis is most prevalent in North African Jews but is reported less frequently in Iraqi Jews, Armenians, and Turks.^18^ In our study, arthritis was diagnosed based on the presence of arthralgia, swelling, and stiffness and was found at 42.8%, which is less frequent than the literature findings. In contrast, arthralgia was more frequent in our patients and was found at 85.7%, similar to a Japanese study of 22 FMF patients, where arthralgia was noted in all patients.^19^ Regarding skin involvement, erysipelas-like erythema is the most common cutaneous feature in FMF occurring in 3–46% of patients,^20^ similar to our finding, where it was found in 11.4% of our patients. In addition, vasculitis such as IgA vasculitis is demonstrated to appear more commonly in FMF patients than in the general population, and its occurrence is thought to be impacted by the presence of *MEFV* gene mutations.^21^ Studies from the Middle East and other countries have revealed the presence of IgA vasculitis in 3.6–7% of patients with FMF.^21,22^ In our study, it was observed in 20% of patients; this high percentage could be attributed to a recruitment bias in our unit receiving severe IgA vasculitis. However, it is difficult to differentiate whether the joint manifestations were associated with IgA vasculitis or FMF since all patients were diagnosed during the IgA vasculitis episode. Therefore, FMF was suspected in patients who maintained high inflammatory markers, abdominal refractory pain to usual treatment, or myositis, with a familial history of consanguinity.

Regarding genetic features, 91.4% of our patients had genomic variants of the *MEFV* gene. *M694V* was the most predominant mutation in our study, which was similarly reported among Arabs, Turks, Armenians, Jews, Germans, and Iranians in previous studies.^23-25^ It is known to be associated with a severe form leading to renal amyloidosis, especially in cases of homozygosity, as well as an early age of symptoms’ onset and a high frequency of arthritis according to Kasifoglu et al.^26^ However, other mutations found in our study, such as *A744S*, *M694I*, and *K695R*, are different from those found in other series, where *E148Q*, *M680I*, and *V726A* are more reported in Arab populations (Table 3).^15,20,23,27^ This mutational diversity of the *MEFV* gene in our study could be attributed to the genetic heterogeneity of the Moroccan population—including Berber Jews autochthons and Arab Muslims—due to the important immigration history of populations. The impact of epigenetic factors may also contribute to this genetic heterogeneity.^27^ Currently, genetic mutations are classified into five groups according to their pathogenic effects, with classes 4 and 5 considered as probably pathogenic or pathogenic, respectively, with the *M694V* mutation belonging to class 5, the most pathogenic. Most of our patients therefore have a mutation of confirmed severe pathogenicity.^32^ The *M694I* mutation is known to be characteristic of the Maghrebian population (Morocco, Algeria, and Tunisia). It appears to be the second most frequent pathogenic variant in the Moroccan and Tunisian populations (33% and 13% respectively) and first in Algeria (80%). It is known to be associated with severe clinical signs.^27^ From a previous Moroccan study, 85% of the patients with *M694I* were Berbers from the Rif region of north-eastern Morocco, suggesting that this variant could be specific to Berber patients and would spread to North African countries in Berber descendants.^33^ However, our two patients with *M694I* had a moderate clinical profile and originated from the west central region of Morocco. The *A744S* mutation appears to be specific to Arab populations, particularly in Lebanon and Egypt with a low frequency and was also reported in one single Ashkenazi Jew patient. It has also been reported in Turkey and Iran.^27^ In our study, this mutation was reported in the heterozygous state in four patients, one of whom had a probable monogenic lupus with *C1Q* deficiency, but however was included in our study in the presence of FMF clinical criteria, the high level of inflammatory markers and response to colchicine. Mutations in the *MEFV* gene have been previously reported in lupus in various studies including *M694V*, *M680I*, *V726A*, and *E148Q*, but to date, no *A744S* mutation has been reported in association with lupus. The presence of *MEFV* gene mutations in patients with monogenic lupus could affect the disease phenotype by increasing inflammatory episodes and lead to genetic FMF tests, which was our case.^34^ However, data about the degree of severity of *A744S* in FMF patients remain to be elucidated. Moreover, the *K695R* mutation was noted in two cases in our study. It was previously reported as a rare variant and was included in the “Infevers” database as a nonfounder mutation in Ashkenazi Jews. It is characterized by a high association with articular manifestations and has an approximative similarity of symptoms with the *P369S* and *M680I* variants.^13^ However, this mutation is common in the central and southeastern European region and has also been reported in Turkey.^35^

The innate immune system is known to be activated by various triggers; evidence suggests that epigenetic factors have an effect on the phenotype of the same *MEFV* variant in the same or different racial groups. In fact, many studies have shown variable severity in FMF according to country of residence, irrespective of ethnicity and pathogenicity of *MEFV* variants.^5^ Ozen et al. showed in the large Eurofever registry of 346 FMF children that patients living in Eastern Mediterranean countries had more severe disease than those living in Western Europe, including Turkish children, suggesting that environmental factors affect FMF phenotypes. Moreover, a multinational study involving 14 countries highlighted a potential environmental origin of amyloidosis susceptibility in FMF, underlining the importance of taking country of residence into account when deciding whether to treat asymptomatic individuals carrying two mutations.^36,37^

In our study, all FMF patients were treated with colchicine with mostly a favorable response (68.6% of cases), characterized by the absence of clinical signs and normalized or reduced inflammatory markers. Our findings are consistent with previous observational studies and randomized controlled trials, where daily colchicine use was associated with complete disease remission and significant improvement in the frequency and severity of FMF attacks (85–90% of patients), as well as amyloidosis prevention. According to the Goldfinger report, colchicine has remained the gold standard and first-line treatment for FMF worldwide since 1972, and it can be administered before the age of one year in children with FMF based on clinical data.^38^

The phenotype–genotype correlation in our study showed a statistically significant difference between patients who had the heterozygous *M694V* mutation (group II) and those with mutations other than *M694V* (group III) regarding the age of onset (p value = 0.037), which was earlier in patients without the heterozygous *M694V* mutation. In contrast to our findings, studies have shown that the *M694V* mutation seems to be associated with an earlier age of onset in FMF, particularly in the homozygous state.^20,39,40^ However, these studies remain controversial.^41,42^ Furthermore, research data underlined the idea that FMF-like symptoms are present in patients with a single variant of the *MEFV* allele despite autosomal recessive transmission of the disease. Indeed, previous studies have described cases of pseudo-dominant transmission of FMF which can be explained by the fact that a heterozygous patient could carry two different variants with only one identified allele.^43^ In Morocco, the most available commercialized kits do not cover all variants, as in the absence of a routine practice next-generation sequencing, the exploration of the *MEFV* gene is limited to exons 10, 2, and 3. This may affect variant characteristics (including age of onset) and interpretation.^24,43^ Recent studies demonstrated that some heterozygous patients may carry severe, high-penetrance pathogenic variants such as *M694V* and *M694I*, which may be present in autosomal dominant inheritance and responsible for severe and early onset phenotypes.^44^ Nevertheless, other studies suggest that additional genes or immune factors may modulate these *MEFV* variant-associated phenotypes.^45^ However, we believe that our result may be attributed to the limited size of our series.

Additionally, a significant difference was noted between groups I and III regarding the frequency of FMF crisis, where it was reported more frequent with homozygous *M694V* patients. Several correlation studies in France, Turkey, and Armenia involving larger cohorts have shown significant frequencies of FMF crisis noted in patients with homozygous mutations in exon 10, particularly *M694V*, compared to other reported genotypes, thus supporting our findings.^24,39,46^ As well, a significant difference was noted between the same groups (I and III) regarding joint involvement, with arthritis being more common in *M694V* homozygous patients (*p* value = 0.005). This finding has been previously demonstrated in several studies comparing homozygous and heterozygous *M694V* genotypes with others; arthritis was an important characteristic of the *M694V* group.^13,20,39,40^ These results are reported in many countries but bring additional data to the characteristics of our Moroccan cohort in the literature, given that there are no phenotype–genotype correlation studies for FMF in our country, apart from a recent study of *MEFV* gene mutations in Moroccan patients with rheumatoid arthritis by Missoum et al.^47^ Nevertheless, there were no other statistically significant differences between the study groups concerning sex ratio, fever, abdominal and chest pain, headache, myalgia, erythema, and IgA vasculitis. This result is similar to that found in previous studies.^14,21^ This study reports the experience of a single Moroccan pediatric rheumatology unit. Although our sample size was small due to the rare and misdiagnosed cases as well as the monocentric type of our study, our results provide an additional argument to the literature, in the absence of current larger studies in the Maghreb and in Morocco, with the aim to expand our sample size in future studies to enable more representative conclusions for our population.

Moreover, the application of a disease severity score could not be applicable to our patients due to the absence of a simplified form in terms of languages suitable for certain patients, as well as the practical difficulty of obtaining a daily form for a follow-up period and the lack of sensitivity to its importance in our social context.

## 5. Conclusion

Our study demonstrated a phenotypic profile similar to previous studies, characterized by the higher frequency of joint involvement and an absence of renal amyloidosis. In addition, we highlight a classical genetic profile with the predominance of homozygous *M694V* mutation, significantly associated with joint involvement but a later onset age. The phenotype–genotype correlation in our study could not be representative of the Moroccan population given the small sample size. We advocate including more patients from other national centers to allow a more conclusive phenotype–genotype correlation for a better management, and a determination of the clinical and genetic characteristics of our population.

## 6. Highlights

– High rate of consanguinity.

– Predominance of the *M694V* mutation in our series, sharing *M694I* among the Maghreb population associated with the Berber origin, and a genetic profile different from neighboring and Arab countries concerning other mutations.

– The absence of amyloidosis despite the high rate of *M694V* homozygosity.

## Abbreviations

**Table:** 

C1Q	Complement component 1q
DNA	Deoxyribonucleic acid
FMF	Familial Mediterranean fever
IgA	Immunoglobulin A
ISSAID	International Society of Systemic Auto-Inflammatory Diseases
MEFV	Mediterranean fever gene
NGS	Next-generation sequencing
OMIM	Online Mendelian Inheritance in Man
PCR	Polymerase chain reaction
PRINTO	Pediatric Rheumatology INternational Trials Organization
UHC	University Hospital Center

## Conflict of Interest Statement

The authors have declared no conflicts of interest.

## Availability of Data and Materials

The data that support the findings of this study are not openly available due to reasons of sensitivity and are available from the corresponding author upon reasonable request.

## Authors’ Contributions

All authors made a substantial contribution to the study and read and approved the final version of the manuscript to be published.

## Figures and Tables

**Table 1.** Mutations identified in our patients.

**Mutation**	**Exon**	**Signification**	**State**	**N**
*M694V*	10	Validated	[Homozygous]	14
			[Heterozygous]	6
*M694I*	10	Validated	[Homozygous]	1
			[Heterozygous]	1
*A744S*	10	Uncertain	[Heterozygous]	4
*K695R*	10	Uncertain	[Heterozygous]	2
*E148Q*	2	Uncertain	[Heterozygous]	1
*R202Q*	2	Validated	[Heterozygous]	1
*G196W*	2	Provisional	[Heterozygous]	1
*P369S/R408Q*	3	Uncertain /Provisional	[Compound Heterozygous]	1

**Table 2.** Clinical features of the subgroups according to the presence of the *M694V* mutation.

	**Homozygous *M694V***	**Heterozygous *M694V***	**Others**	***p* value—Signification**					
	**Group I (n=14)**	**Group II (n=6)**	**Group III (n=12)**	**Group I and II**	**OR [Confidence interval]**	**Group I and III**	**OR [Confidence interval]**	**Group II and III**	**OR [Confidence interval]**
Male/female	9/5	1/5	5/7	0.141	0.111 [0.010–1.236]	0.431	0.397 [0.081–1.936]	0.6	3.571 [0,313–40.751]
Age of onset	6.2 ± 3.2	9 ± 5.8	3.9 ± 3.7	0.188	-	0.09	-	0.037	-
Positive family history	8 (57.1%)	2 (33.3%)	2 (16.7%)	0.628	0.375 [0.051–2.772]	0.051*	0.150 [0.024–0.955]	0.569	0.400 [0.041–3.900]
			a : 0						
			b : 9						
	a : 4 (28.5%)	a : 2 (33.3%)	(75%)						
Frequency***	b : 5 (35.7%)	b : 0	c : 1	0.485	-	**0.028**	-	0.07	-
	c : 5 (35.7%)	c : 2 (33.3%)	(8.3%)						
			d : 1						
			(8.3%)						
Fever	13 (9.9%)	5 (83.3%)	9 (75%)	0.316	1.200 [0.839–1.716]	0.096	1.333 [0.962–1.848]	1	0.600 [0.049–7.408]
Chest pain	5 (37.5%)	4 (66.7%)	4 (33.3%)	0.336	3.600 [0.478–27.110]	1	0.900 [0.177–4.564}	0.321	0.250 [0.031–1.999]
Headache	1 (7.1%)	1 (16.7%)	2 (16.7%)	0.521	2.600 [0.135–50.049]	0.58	2.600 [0.205–32.904]	1	1 [0.072–13.868]
Arthralgia	9 (64.3%)	6 (100%)	12 (100%)	0.26	0.643 [0.435–0.950]	**0.042**	0.643 [0.435–0.950]	- **	-
Arthritis	11 (78.6%)	4 (66.7%)	2 (16.7%)	0.613	0.545 [0.065–4.562]	**0.005**	0.055 [0.008–0.396 ]	0.107	0.100 [0.010–0.975]
Myalgia	1 (7.1%)	1 (16.7%)	0	0.521	2.600 [0.135–50.049]	1	0.929 [0.803–1.074]	0.333	0.833 [0.583–1.192]
Abdominal pain	13 (92.9%)	4 (66.7%)	11 (91.7%)	0.202	0.154 [0.011–2.176]	1	0.846 [0.047–15.161]	0.245	5.500 [0.385–78.573]
Erysipelas-Like Erythema	5 (35.7%)	4 (66.7%)	4 (33.3%)	0.336	3.600 [0.478–27.110]	1	0.900 [0.177–4.564]	0.321	0.250 [0.031–1.999]
IgA Vasculitis	4 (28.6%)	1 (16.7%)	1 (8.3%)	1	0.500 [0.044–5.737]	0.33	0.227 [0.022–2.390]	1	0.455 [0.023–8.829]
Response to treatment****	t : 11 (78.5%)	t : 3 (50%)	t : 7 (58.3%)	0.579	-	0.367	-	0.689	-
	p : 2 (14.3%)	p : 1 (16.6%)	p : 3 (25%)						

*p* value <0.05.

* Close to significance value.

** No statistics were calculated because “Arthralgia” is considered a constant value.

*** Frequency: a: <1 time/month. b: 1-2 times/month. c: >2 times/month. d: prolonged fever.

**** Response to treatment: t: total. p: partial.

**Table 3.** Comparison of clinical and genetic features of FMF patients in other countries.

	**Our study**	**Maroc^30^**	**Algeria^31^**	**Tunisia^17^**	**Egypt^16^**	**Syria^21^**	**Turkey^19^**	**Eurofever^32^**	**AID-Net^27^**	**Armenia^33^**	**Japan^20^**
		**2012**	**2011**	**2007**	**2020**	**2014**	**2019**	**2017**	**2013**	**2018**	**2020**
Male/female	17/18	52/68	35/36	76/63	46/49	52/51	207/220	346	132/110	5489/4881	9/13
Consanguinity	54.3%	60%	41%	-	37.8%	-	5.6%	-	ND	-	-
Familial case	34.3%		61%	19%	22.1%	31 %	42.7%	-	-	33.9%	31.8%
Fever	**74.3%**	**90%**	**97%**	-	96.8%	79.6%	85.4%	**95.9%**	**80.6%**	92.3%	100%
Abdominal pain	**83%**	**88%**	**85%**	-	94.7%	88.3 %	95.1%	**87.2%**	**75.6%**	86.5%	54.5%
Chest pain	34.3%	36%	26%	-	17.8%	16.4%	1.4%	47.9%	10.7%	48.4%	36.4%
Joint involvement	**100%**	**62%**	**59%**	-	78.9%	26.2%	**69.2%**	**84.1%**	24%	16.8%	45.5%
Skin involvement	71.4%	-	-	-	20%	4.8%	3.5%	69.9%	4.5%	14.8%	13.6
AA amyloidosis	0	0.8%	8%	3.6%	0	0	0	0.5%	0	0.6%	0
Colchicine	100%	100%	-	96.4%	100%	31%	100%	97.9%	25%	-	100%
Favorable response	68.6%	54%	-	-	23.1%	75%	72.3%	77.7%	ND	-	95.5%
FMF Mutations	*M694V* (62.5%)	*M694V* (47%)	*M694I* (50%)	*M680I* (32%)	*E148Q* (25.2%)	*M694V* (36.4%)	*M694V* (41.5%)	*M694V* (80.6%)	*M694V* (55.2%)	*M694V* (41.3%)	*E148Q* (54.5%)
	*A744S* (12.5%)	*M694I* (32%)	*M694V* (14%)	*M694V* (27%)	*V726A* (20%)	*E148Q* (14.9%)	*E148Q* (38.1%)	*V726A* (20.1%)	*M680I* (11.8%)	*V726A* (27.6%)	*P369S* (31.8%)
	*M694I* (6.2%)	*A744S* (6.5%)	*E148Q* (12%)	*E148Q* (18%)	*M680I* (20%)	*M680I*(G/C) (14%)	*M680I* (26.4%)	*M680I* (G>C) (18%)	*V726A* (10%)	*M680I* (18.1%)	*R408Q* (27.2%)
	*K695R* (6.2%)	*M680L* (4%)	*A744S* (10%)	*M694I* (13%)	*M694V* (17.8%)	*M694I* (11.6%)	*V726A* (22.5%)	*E148Q* (13%)	*G148G* (7.9%)	*E148Q* (5.3%)	*M694I* (22.7)
	*E148Q* (2.8%)	*M694del* (2%)	*M680I* (8%)	*V726A* (5%)	*M694I* (7.3%)	*V726A* (10.7%)	*P369S* (16.9%)	*R202Q* (5.4%)		*R761H* (3.4%)	*L110P* (13.6%)
	*R202Q* (3.1%)	*E148Q* (6.5%)	*I692del* (2%)	*A744S* (3%)	*P369S* (1%)	*R761H* (5%)	*A744S* (12.5%)	*A744S* (2.9%)		*F479L* (2.5%)	*R202Q* (9%)
	*G196W* (3.1%)		*I591T* (2%)	*I692del* (1%)	*A744S* (1%)	*A744S* (2.5%)	*R761H* (2.1%)	*P369S* (2.9%)		*P369S* (0.5%)	*G304R* (4.5%)
	*P369S/R408Q* (3.1%)		*P369S* (2%)	*R761H* (1%)	*M762V* (1%)	*K695R* (1.7%)		*K695R* (1.6%)		*M694I* (0.4%)	*S503C* (4.5%)
						*M680I*(G/A) (0.8%)				*A744S* (0.3%)	*3’UTR variant* (4.5%)
						*P369S* (0.8%)				*K695R* (0.1%)	
						*F479L* (0.8%)					
						*I692del* (0.8%)					
Homozygous	46.8%	20%	67.2%	45.9%	25.26%	18.9 %	18%	34.5%	38.4%	16.2%	4.5%
Heterozygous	50%	-	16.8%	27.8%	66.32%	44.6 %	52%	27.9%	32.2%	25.3%	9%
Compound heterozygous	3%	10.8%	15.4%	26.2%	8.42%	36.5 %	22%	40.9%	25.2%	58.2%	72.7%
